# Metagenome-assembled genomes from particle-associated microbial communities in the mesopelagic zone of the Pacific Ocean

**DOI:** 10.1128/MRA.00614-23

**Published:** 2023-11-22

**Authors:** Jacqueline Hollensteiner, Dominik Schneider, Anja Poehlein, Axel Himmelbach, Rolf Daniel

**Affiliations:** 1 Genomic and Applied Microbiology and Göttingen Genomics Laboratory, Institute of Microbiology and Genetics, Georg-August University of Göttingen, Göttingen, Germany; 2 Leibniz Institute of Plant Genetics and Crop Plant Research (IPK), Gartersleben, Leipzig, Germany; Montana State University, Bozeman, Montana, USA

**Keywords:** metagenome-assembled genomes (MAGs), Pacific Ocean, particle-associated lifestyle, mesopelagic zone, biogeochemical cycling

## Abstract

We report 10 particle-associated metagenome-assembled genomes (MAGs) from the mesopelagic zone of Pacific Ocean seawaters. MAGs comprise members of *Flavobacteria Halomonas*, *Blastomonas*, *Brevundimonas*, *Alteromonas, Shingomonas*, *Sphingopyxis*, *Tabrizicola, Proteobacteria*, and *Gammaproteobacteria*. Functional annotation suggests that these bacteria are involved in central particulate organic carbon conversion, nitrogen cycling, and phosphorus cycling.

## ANNOUNCEMENT

The metagenome-assembled genomes (MAGs) derived from a water sample were employed for the investigation of the particle-associated microbial community of a mesopelagic zone in the Pacific Ocean.

The water sample was collected in May 2016 at the SONNE research vessel cruise SO248 (BacGeoPac) in the Pacific Ocean (28 °N; 177.33 °E) at a depth of in 300 m. Approximately 10 L were filtered through a 20-µM nylon net (Millipore, Billerica, MA, USA) and through a 2.7-µM glass fiber filter (Whatman GF/D; GE Healthcare, Little Chalfont, UK) to investigate the particle-associated microbial community. The filter was kept at −80°C until further usage. The total community DNA was extracted according to Hollensteiner et al. (1). Illumina pair-end sequencing libraries were prepared and indexed using the Nextera DNA Flex library preparation kit and the Nextera DNA CD index kit as recommended by the manufacturer (Illumina, San Diego, CA, USA). Sequencing was performed with an Illumina NovaSeq 6000 system, the NovaSeq 6000 SP Reagent Kit v1.5 (paired-end mode with 2 × 250 bp cycles), and the NovaSeq XP 2-Lane Kit (v1.5 chemistry).

Default software parameters were used unless otherwise stated. Sequencing resulted in 119.2 million total reads for the whole metagenome sample. Sequence data processing comprised fastp v.0.20.1 (2) with overlap correction, quality filtering (removal of reads < Q20), read clipping with a sliding window of 4, removal of reads shorter than 50 bp, and Illumina adapter removal. Quality-filtering resulted in 113.2 million paired-end reads and 24.5 billion bp (average read length 216 bp), which were assembled using metaSPAdes v3.14.1 (3). The assembly yielded 113,938 contigs > 1 kb with a total length of 227,862,326 bp and an average GC content of 53.4% calculated with MetaQUAST v5.0.2 (4). VSEARCH v2.15.0 (5) was used for setting a contig length cutoff (--minseqlength 1,000). Reads were mapped and indexed using Bowtie2 v.2.4.1 (6) and SAMtools v1.9 (7). Coverage was calculated using Qualimap v2.2.2d (8). Paired-end reads were filtered from sorted bam files by using the fastq command of bowtie2 v.2.4.1 (6). Metagenome-assembled genomes were generated using MetaBat2 v2.15 (9). Completeness and redundancy were assessed using CheckM v1.1.2 (10); bins with <75% completeness and >5% contamination were subjected to annotation via Prokaryotic Genome Annotation Pipeline (PGAP) (11). Taxonomic identification of MAGs was performed with GTDB-Tk v1.3.0 (12), employing “classify_wf.” The metabolic potential was analyzed using GhostKOALA (13). KEGG Decoder v1.2.1 (14) was used to determine the presence or absence of metabolic pathways.

**Fig 1:**
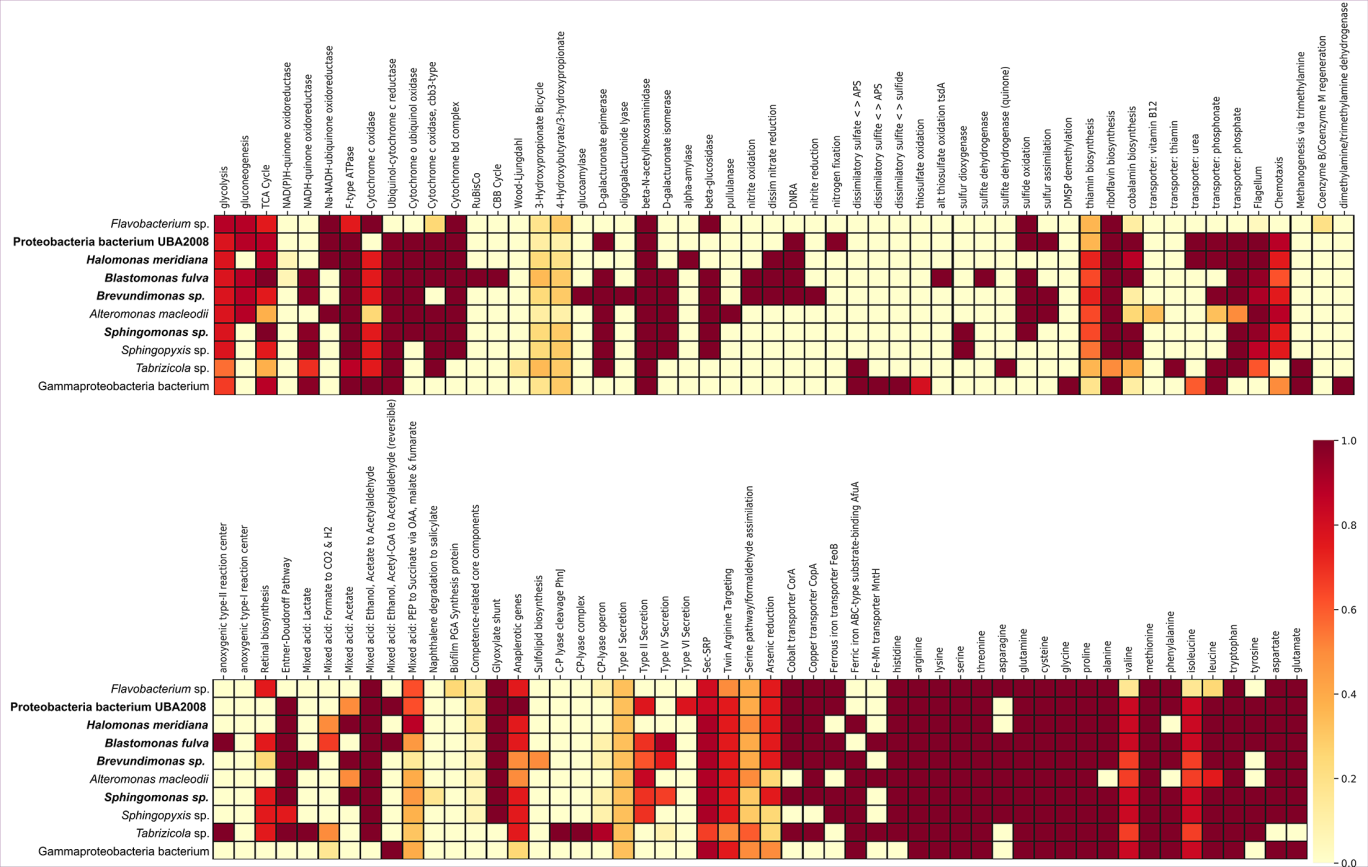
Heatmap of metabolic functions of 10 particle-associated MAGs. The heatmap represents metabolic pathway completeness based on the presence or absence of genes determined by KEGG Decoder v.1.2.1. The color gradient is depicted on the right and reflects the fractional abundance of genes associated with a pathway encoded by a particular genome. High-quality MAGs are marked in bold, and missing pathways were removed.

In total, five bacterial high-quality (bold) and five medium-quality MAGs were retrieved (15). MAG statistics are summarized in Table 1. Taxonomic analyses revealed MAGs only from Gram-negative bacteria belonging to 10 different taxa, including *Tabrizicola*, *Sphingomonas*, *Flavobacterium*, *Blastomonas*, *Halomonas, Sphingopyxis*, *Brevundimonas* and *Alteromonas,* while two could only be classified as Proteobacterium and Gammaproteobacterium. The high diversity of MAGs indicates that the variety of bacteria contributes to maintaining biogeochemical cycling in the twilight zone. The functional analysis revealed genes involved in various remineralization processes (Fig. 1), including central particulate organic carbon conversion (glycolysis, TCA cycle, NADH-quinone oxidoreductases, anoxygenic type II reaction center), nitrogen cycling, including nitrate reduction, N_2_ fixation, ammonium oxidation, nitrite reduction, and phosphorus cycling (C-P lyase complex). In addition, heterotrophic as well as photoheterotrophic lifestyles were encoded by different MAGs.

**TABLE 1:** Features of MAGs from the Pacific Ocean^*a*^

Bin	GTDB-Tk genus classification	BioSample	Compl./ cont.	Size (bp)	No. of contigs	No. of CDSs	N50 (bp)	GC content (%)	Fastani ANI (%)	Genome accession no.	Mean coverage
**Bin02**	**Proteobacteria bacterium UBA2009**	**SAMN20244328**	**98.18/0.84**	**4,079,811**	**28**	**3,706/3,697**	**251,278**	**55.9**	**N/A**	**JAIXHJ000000000**	**71**
Bin05	*Tabrizicola* sp.	SAMN20244329	75.38/1.50	2,453,108	486	2,777/2,468	5,427	66.3	98.25	JAIXHK000000000	16
**Bin06**	***Sphingomonas* sp**.	**SAMN20244330**	**99.52/2.72**	**4,658,187**	**82**	**4,554/4,506**	**160,357**	**65.6**	**N/A**	**JAIXHL000000000**	**48**
Bin08	*Flavobacterium* sp.	SAMN20244331	95.69/5.49	2,830,155	288	2,886/2,654	14,178	30.7	N/A	JAIXHM000000000	16
**Bin13**	***Blastomonas fulva***	**SAMN20244332**	**99.37/2.99**	**4,797,095**	**264**	**4,709/4,542**	**31,927**	**63.8**	**97.42**	**JAIXHN000000000**	**19**
Bin14	Gammaproteobacteria bacterium	SAMN20244333	93.60/2.44	2,971,028	229	2,977/2,769	19,848	51.3	99.53	JAIXHO000000000	108
**Bin_18**	***Halomonas meridiana***	**SAMN20244334**	**99.57/0.43**	**3,462,055**	**83**	**3,284/3,216**	**69,917**	**57.0**	**97.15**	**JAIXHP000000000**	**295**
Bin_28	*Sphingopyxis* sp.	SAMN20244335	94.03/1.19	2,829,893	89	2,662/2,600	92,432	64.0	N/A	JAIXHQ000000000	46
**Bin_29**	***Brevundimonas* sp**.	**SAMN20244336**	**100/0.32**	**3,499,373**	**32**	**3,360/3,327**	**311,796**	**67.0**	**N/A**	**JAIXHR000000000**	**53**
Bin_31	*Alteromonas macleodii*	SAMN20244330	85.48/2.60	4,032,519	496	3,860/3,425	10,630	44.5	97.12	JAIXHS000000000	48

^*a*^ High-quality MAGs are displayed in bold.

## Data Availability

The raw paired-end sequences of the metagenome have been deposited in the NCBI Sequence Read Archive with the accession number SRR24425160 under the BioProject PRJNA746715 and BioSample accession number SAMN20356034. MAGs have the same BioProject number PRJNA746715 and can be found under BioSample SAMN20244328-SAMN20244337 with genome accession numbers JAIXHJ000000000-JAIXHS000000000.
